# The effect of immersive virtual reality on proximal and conditioned threat

**DOI:** 10.1038/s41598-019-53971-z

**Published:** 2019-11-22

**Authors:** Jörgen Rosén, Granit Kastrati, Aksel Reppling, Klas Bergkvist, Fredrik Åhs

**Affiliations:** 1 Department of Psychology, Uppsala University, Uppsala, Sweden; 2 Department of Clinical Neuroscience, Karolinska Institutet, Stockholm, Sweden; 3 Department of Psychology and Social Work, Mid Sweden University, Östersund, Sweden

**Keywords:** Emotion, Human behaviour

## Abstract

Virtual reality lets the user be immersed in a 3-dimensional environment, which can enhance certain emotional responses to stimuli relative to experiencing them on a flat computer screen. We here tested whether displaying two different types of threats in immersive virtual reality enhanced threat related autonomic responses measured by skin conductance responses (SCRs). We studied innate and learned threat responses because these types of threats have been shown to depend on different neural circuits in animals. Therefore, it is possible that immersive virtual reality may modulate one of these threats but not the other. Innate threat responses were provoked by the sudden appearance of characters at proximal egocentric distance, which were compared to the sudden appearance of distant characters (proximal threat). Learned threat responses were studied by conditioning two of the characters to an electric shock (conditioned threat) and contrasting SCRs to these characters with SCRs to two other characters that were never paired with shock. We found that displaying stimuli in immersive virtual reality enhanced proximal threat responses but not conditioned threat responses. Findings show that immersive virtual reality can enhance an innate type of threat responses without affecting a learned threat response, suggesting that separate neural pathways serve these threat responses.

## Introduction

The ability to detect threat in the environment is important for survival. Because threat detection enhances survival, and hence the ability to get offspring, specialized neural circuits have evolved to manage speedy monitoring of dangers. Threat detection that depends on evolutionary pressure and does not require prior experience with the feared stimulus to evoke a defensive response is considered innate. An example of innate threat is proximal threat, which is threat that is perceived when objects appear suddenly near the body or when personal space is intruded upon^1–5^. This effect can be observed in infants where the unexpected appearance of proximal objects triggers an innate threat response^6^. Defensive responses to proximal encounters have received a great deal of attention in animal literature. Here, the intensity of defensive behaviors have been found to be modulated by distance to threat^7^. A possible explanation is that proximal threats necessitate a more urgent response as compared to distal threats, thus increasing the likelihood of survival behaviors. Similar results have also been obtained in humans, where it has been demonstrated that subjective degree of anticipatory pain increases as proximity to threat increases^8^.

In contrast to innate threat, learned threat depends on prior opportunities of learning before fear can be acquired. A common and well-studied type of learned threat is fear conditioning, which is developed through repeated exposures to a neutral stimulus that predicts an aversive outcome. For example, learning that an electrical shock is associated with a neutral stimulus results in expression of fear to the this stimulus^9^.

At the neural level, innate and conditioned threat responses depend on different visual processing streams. For proximal threat detection, areas early on in the visual stream are important, such as the superior colliculus^10^. In turn, these areas project to regions further downstream that are important for initiating defensive responses, such as amygdala nuclei^11^ and the periaqueductal gray^8^. When considering detection of conditioned fear cues, areas such as the lateral geniculate body and the lateral posterior nucleus of the thalamus are more dominant^12^. Similar to innate threat, the amygdala circuitry is also important for initiating defensive responses to conditioned threat^13^. These previous findings suggest that different neural systems support proximal and conditioned threat, but why these brain pathways are selective to different threats is not understood. In other words, why is fear not detected and expressed through one common neural pathway? We hypothesized that one reason could be that different threats are detected based on different types of visual information. Thus, stimulus qualities may modulate the defensive response because of divergence in neural circuitry responsible for detection and expression of fear. This would be in line with findings in animal studies that demonstrated a division of neural circuitry for different types of threats^14^. A way to test this would be to test if it is possible to modulate one type of threat response without affecting the other. This would speak to the idea that there are separate pathways supporting different types of threat.

In fear research, the use of virtual reality headsets has become increasingly popular. This novel technology is useful when studying defensive responses to threats in humans, especially when investigating defensive responses that depends on egocentric distance to threats^2,4,15^. One key difference between displaying stimuli in virtual reality compared to a computer display is that the visual experience becomes immersive. Immersion is defined by the degree of which sensory systems in the body are stimulated by the physical world. In a head-mounted display, immersion is primarily achieved by improved depth perception and a larger visual field compared to a traditional computer display, essentially allowing for a richer visual experience^16^.

Immersive virtual-reality differs from traditional computer displays in that different images are presented to the left and right eye to enhance depth perception through stereopsis. Stereopsis refers to the ability to distinguish the relative distance of objects by their apparent physical displacement^17^. The slight difference in projection of the images on the retina for each eye is what makes stereoscopic depth discrimination possible. This technique is commonly used in a head-mounted display to simulate depth to induce a sense of immersion in the virtual environment. How immersive virtual-reality changes emotional responses to stimuli is largely unexplored.

**Figure 1:**
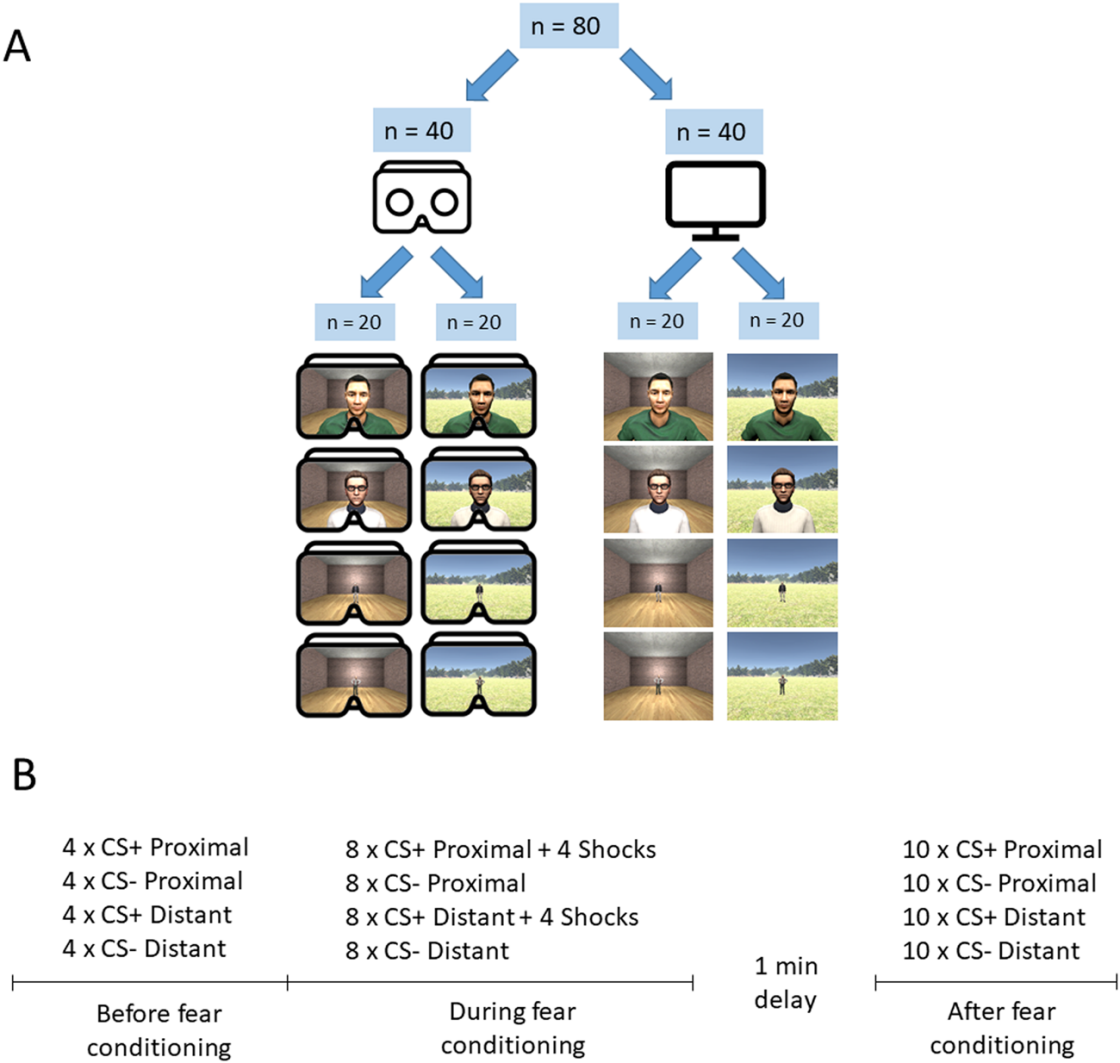
Study design with virtual character presentations (**A**) and timeline (**B**). A total of 80 participants were divided into two groups; one using an immersive virtual-reality head-mounted display (**A**, left) and the other using a computer display (**A**, right). Four different virtual character presentations were used for the stimuli presentations (all male, two shown in **A**) with Indoor and Outdoor environments serving as conditioning contexts.

In this study, we investigated whether autonomic responses to proximal and conditioned threat were modulated by immersive virtual-reality. We compared autonomic responses in one group of participants that viewed threat cues displayed on a computer screen to another group of participants that viewed threat cues in an immersive virtual-reality head-mounted display. For both display media, four virtual characters were presented at proximal and distant egocentric distances. One proximal and one distant virtual character served as conditioned threat cues (CS+) and were paired with a mild electric shock, while the other two served as distance matched control cues (CS−) and were never paired with a shock (Fig. 1). Autonomic responses to stimuli presentations were indexed by measuring skin conductance responses (SCR). Since studies demonstrated that proximal encounters constitute a greater threat compared to distant ones and induce stronger intensity in defensive behaviors^7,8,18^, we expected increased SCR to proximal compared to distant stimuli presentations. In addition, we hypothesized that presenting visual threat cues in a head-mounted display would result in greater SCR for proximal but not conditioned threat compared to a computer display. This would indicate a difference in the visual processing of proximal and conditioned threat, which could be a result of the division of neural pathways processing these threats that has been reported in animals^14,19^.

## Results

### Threat responses before conditioning

Before fear conditioning, we observed greater SCR for proximal compared to distant presentations of virtual characters across both display types (immersive virtual-reality head-mounted display and computer display) (Distance: *F*_1, 80_ = 178.92, *p* < 0.001, *η*^2^ = 0.70). However, SCR to proximal compared to distant presentation of virtual characters were greater when displayed in immersive virtual reality head-mounted display than computer display (Distance x Display: *F*_1, 80_ = 22.28, *p* < 0.001, *η*^2^ = 0.23). We found no effect of Context or any interaction between Display type and Context (Indoor, Outdoor) and no main effect of display (all *p*’s > 0.14). See Table 1 for SCR means and standard deviations and Supplemental Table 1 for ANOVA statistics.

**Table 1:** Mean range corrected SCR (SD) before, during and after fear conditioning.

		HMD				2D			
		Proximal		Distant		Proximal		Distant	
		CS+	CS−	CS+	CS−	CS+	CS−	CS+	CS−
Before	Indoor	0.49 (0.17)	0.52 (0.15)	0.30 (0.16)	0.33 (0.16)	0.37 (0.17)	0.40 (0.16)	0.33 (0.16)	0.30 (0.14)
	Outdoor	0.43 (0.10)	0.46 (0.16)	0.30 (0.12)	0.28 (0.11)	0.41 (0.19)	0.40 (0.17)	0.32 (0.16)	0.30 (0.12)
During	Indoor	0.48 (0.17)	0.46 (0.16)	0.43 (0.19)	0.34 (0.15)	0.41 (0.17)	0.39 (0.17)	0.38 (0.16)	0.32 (0.14)
	Outdoor	0.42 (0.15)	0.44 (0.11)	0.35 (0.11)	0.30 (0.09)	0.43 (0.18)	0.39 (0.15)	0.41 (0.16)	0.33 (0.13)
After	Indoor	0.39 (0.19)	0.37 (0.16)	0.27 (0.11)	0.26 (0.13)	0.32 (0.19)	0.30 (0.22)	0.31 (0.18)	0.24 (0.16)
	Outdoor	0.28 (0.19)	0.26 (0.15)	0.23 (0.13)	0.19 (0.11)	0.34 (0.18)	0.30 (0.16)	0.30 (0.16)	0.28 (0.15)

Means for groups using an immersive virtual-reality head-mounted display (HMD) and computer display (2D) are tabulated separately.

### The effect of immersive virtual reality on proximal and conditioned threat

**Figure 2:**
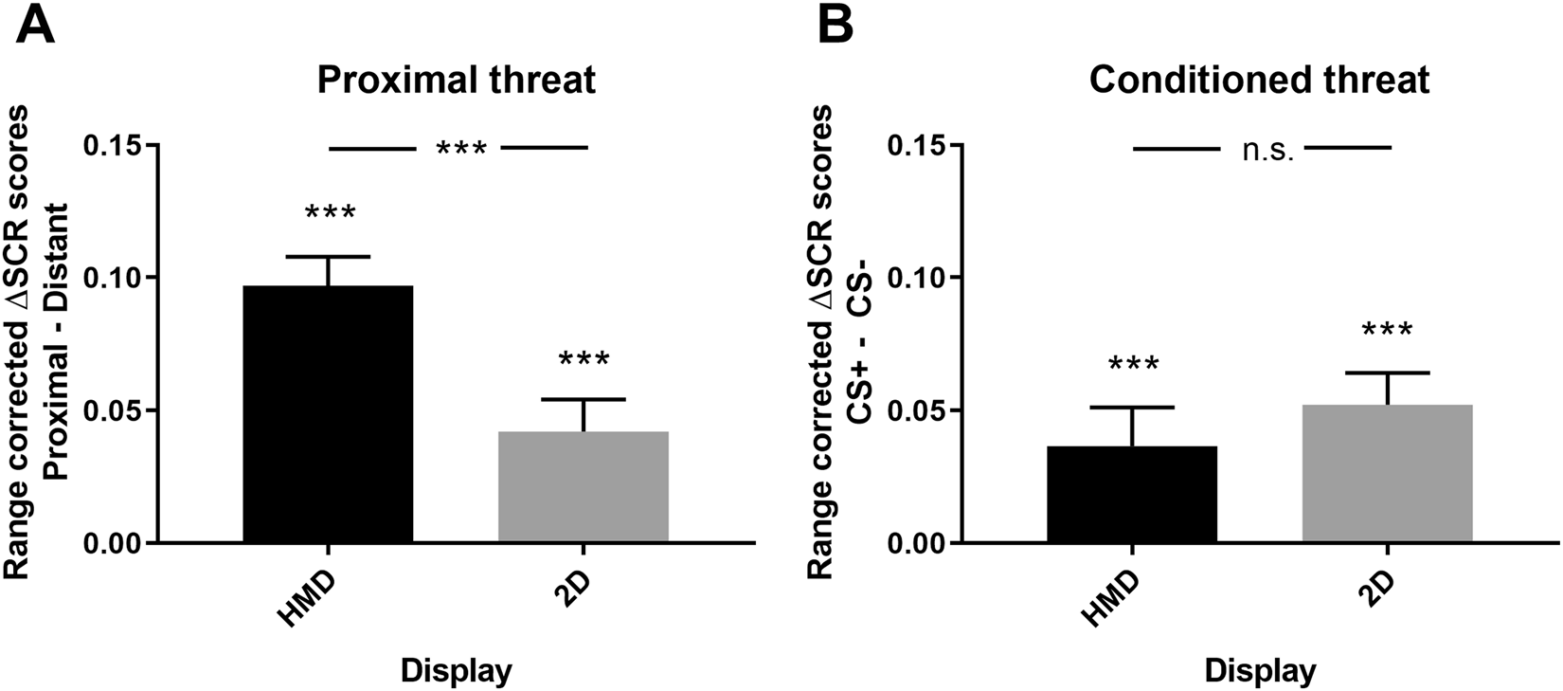
Effect of display type on proximal and conditioned threat responses. (**A**) The mean difference in threat responses to proximal relative to distant CSs was greater when CSs were presented in an immersive virtual-reality head-mounted display (HMD) than when presented on a computer screen (2D). (**B**) For conditioned threat, the mean difference between CS+ and CS− was similar when CSs were presented in an immersive virtual-reality head-mounted display (HMD) as when presented on a computer display (2D). Error bars show standard error of measurement. CS+, Conditioned stimulus; CS−, control stimulus; n.s., non-significant; ***p < 0.001.

As before conditioning, we observed greater SCR to proximal compared to distant presentations of virtual characters (Distance: *F*_1, 80_ = 72.83, *p* < 0.001, *η*^2^ = 0.49). Importantly, we found SCR to be greater for proximal relative to distant characters when we compared immersive virtual reality head-mounted display to computer display (Distance x Display: *F*_1, 80_ = 11.35, *p* < 0.001, *η*^2^ = 0.13) (see Fig. 2). We observed greater SCR for proximal compared to distant stimuli for both immersive virtual reality head-mounted display (*t*_1,40_ = 8.15, *p* < 0.001) and computer display (*t*_1,40_ = 4.43, *p* < 0.001) (see Fig. 2A). During conditioning, SCR was greater to CS+ presentations compared to CS− presentations (CS: *F*_1, 80_ = 21.92, *p* < 0.001, *η*^2^ = 0.24) indicating successful conditioning. SCR was greater for CS+ over CS− in both the immersive virtual reality head-mounted display (*t*_1,40_ = 3.91, *p* < 0.001) and the computer display (*t*_1,40_ = 4.45, *p* < 0.001) (see Fig. 2B). The difference in SCR between CS+ and CS− was similar in immersive virtual reality head-mounted display and on the computer display as there was no interaction between display type and CS-type (CS x Display: *F*_1, 80_ = 0.68, *p* = 0.41, *η*^2^ = 0.01). Additionally, we discovered less CS-differentiation to proximal relative to distant stimuli presentations (CS x Distance: *F*_1, 80_ = 15.53, *p* < 0.001, *η*^2^ = 0.17). The effect of Distance (Proximal, Distant) or Conditioning (CS+, CS−) was not modulated by Context (all *p*’s > 0.19), indicating that results generalized across the two virtual reality environments (Indoor, Outdoor) used in our study. See Table 1 for SCR means and standard deviations and Supplemental Table 2 for ANOVA statistics.

### Proximal and conditioned threat after conditioning

Following conditioning, we again observed greater SCR to proximal compared to distant CS presentations (Distance: *F*_1, 80_ = 40.74, *p* < 0.001, *η*^2^ = 0.35). In addition, proximal threat responses were greater in immersive virtual reality head-mounted display compared to computer display (Distance x Display: *F*_1, 80_ = 9.84, *p* = 0.002, *η*^2^ = 0.12) (see Supplemental Fig. 1). We found greater SCR to the CS+ compared to the CS− (CS: *F*_1, 80_ = 18.11, *p* < 0.001, *η*^2^ = 0.19), but no reduction in CS-discrimination to proximal CSs as was observed during conditioning (CS x Distance: *F*_1, 80_ = 0.55, *p* = 0.1, *η*^2^ = 0.46). See Table 1 for SCR means and standard deviations and Supplemental Table 1 for ANOVA statistics. Extinction of conditioned threat occurred as expected as CS discrimination diminished with time (CS x Time: *F*_1, 80_ = 10.69, *p* = 0.002, *η*^2^ = 0.12). In contrast, no habituation of proximal threat occurred as a function of time (Distance x Time: *F*_1, 80_ = 2.49, *p* = 0.119, *η*^2^ = 0.03).

## Discussion

In this study, we examined whether immersive virtual-reality modulates proximal and conditioned threat responses indexed by SCR. The results indicate that presenting stimuli in immersive virtual-reality facilitates SCR to proximal threat cues. This facilitating effect of immersive virtual reality on SCR was not observed for conditioned threat cues. Thus, immersive virtual reality modulates proximal but not conditioned threat. A possible explanation for this finding is that neural circuits selective for proximal threat are sensitive to differences in how visual information is presented in a head-mounted display relative to a flat computer screen whereas circuits serving conditioned threat are not.

Our finding of a modulation of innate (proximal) but not conditioned threat by immersive virtual-reality in a head-mounted display indicates a difference in neural processing between innate and conditioned threat. The idea that innate and conditioned threat is processed separately has been demonstrated in animal studies^14,19^ and one of our previous studies has tested this hypothesis in humans^4^. In our previous study, we used images of 3-dimensional inanimate objects (spheres) and social stimuli (virtual characters) to test whether social content influenced proximal and conditioned threat responses using a virtual-reality head-mounted display. As in the present study, we observed greater SCR to proximal compared to distant stimuli. This effect was modulated by social content as proximal characters evoked greater SCR than proximal spheres. Social content did however not influence conditioned threat responses, indicating a separation of pathways governing innate and conditioned fear in the human brain.

The finding that neither social content^4^ nor immersive virtual-reality enhance fear conditioning or slow extinction is also consistent with conditioning studies that have used fear-relevant conditioned cues. These studies have tested a prediction of preparedness theory^20^. Preparedness theory posits that associative memories between stimuli that have been a threat to survival in our evolutionary past and a fearful experience are difficult to extinguish. According to preparedness theory, this could explain why spider and snake phobias are more prevalent than phobias of guns, although guns are more life threatening than snakes or spiders in the urbanized world of today. However, a systematic review of studies that have used fear-relevant animals as conditioned cues show that the majority of published experiments have not found that responses conditioned to fear-relevant stimuli are more difficult to extinguish than responses conditioned to stimulus classes that are neutral, such as mushrooms and flowers^21^. A lack of modulation of extinction of conditioned fear fits with the idea that separate functions support fear of snakes and spiders on the one hand and conditioned fear on the other.

The modulation of proximal threat by immersive virtual-reality in a head-mounted display suggests that pathways selective for proximal threat detection are sensitive to this type of manipulation of visual information. One obvious difference between displaying stimuli in a virtual-reality head-mounted display relative to a computer display is that 3-dimensional depth cues can be better perceived in a head-mounted display due to the stereoscopic presentation mode. It is then possible that brain circuits processing proximal threat are also sensitive to stereoscopic presentations of stimuli. A region in the brain found to be important for processing proximal threat is the superior colliculus^10^. The superior colliculus is a map like structure, responsible for processing a multitude of visual, auditory and somatosensory modality-specific stimulus selectivities^22^. This structure has demonstrated increased activity when processing depth related information through a feedback process from the intraparietal sulcus^23^. Further evidence to the importance of superior colliculus for visual depth processing can be found in animal lesion studies. Findings here indicate that a compromised superior colliculus impairs performance on proximal reaching tasks when the cues are binocular but not monocular^24^. In addition, studies shown that the superior colliculus is important for fine tuning visuo-tactile operations in proximal space and coordinating hand-eye movements proximal to the body^25,26^. This eye-muscle coordination is what makes stereoscopic vision possible. Thus, it can be argued that the superior colliculus is important for both processing proximal threat cues and depth information, suggesting that a modulation of proximal threat by depth information could occur in the superior colliculus. Further, a brain-imaging study found differences in activity of higher order visual areas in the parietal cortex when comparing stereoscopic and monoscopic images^27^. In addition, areas early in the visual stream such as the superior colliculus seem to respond less strongly when only one eye is used to process visual stimuli^28^, suggesting a selectivity for binocular visual input which is required for depth perception. In summary, it seems that neural computation supporting depth perception is implemented both at early and later stages of the visual processing hierarchy and could influence proximal threat responses.

Another important finding in our study was that monoscopic depth cues were sufficient for triggering proximal threat responses. This was evident as participants that viewed characters displayed on a computer monitor also exhibited elevated SCR to proximal as compared to distal presentations. The result could indicate that visual pathways that are not sensitive to visual depth information also could contribute to proximal threat responses. Evidence suggests that neural responses in the lateral geniculate nucleus are sensitive to monoscopic depth cues in cats and primates^29^. While the lateral geniculate nucleus is responsible for integration of binocular information from both eyes^30,31^, findings indicate an insensitivity to stereoscopic depth cues^32^. This suggests that the lateral geniculate nucleus is important for evaluating proximal threat relying on monoscopic depth cues.

In rodents, defensive behaviors to innate types of threats are suggested to be dependent on distinct outputs from the amygdala, routed through neural pathways around the brainstem^14^. This has also been demonstrated in non-human primates where pharmacological inhibition of the basolateral amygdala was found to decrease the occurrence of defensive behaviors (i.e. escaping) during proximal social interactions^11^. Findings in humans also suggest that the amygdala and regions around the brainstem is important for regulating defensive responses to proximal threats. For example, one imaging study found that increased fear in response to a proximal tarantula was associated with activation in the amygdala, bed nucleus of the stria terminalis and the periacqueductal grey^33^. In addition, one recent report, using imaging and tractography, found evidence for a rapid response pathway to visual threats (i.e. proximal faces) routed from the superior colliculus via the pulvinar to the amygdala^34^. These findings suggests that the amygdala, together with areas around the brainstem, are important for orchestrating defensive responses to proximal threats.

Anatomical and behavioral studies have demonstrated that visual pathways routed through the lateral geniculate body and the lateral posterior nucleus of the thalamus is important for processing of conditioned threat^12^. Further downstream, defensive circuitry such as the amygdala found to be responsible for facilitating the proper responses^35^. This suggests that processing of proximal and conditioned threat diverges in early response pathways but converges at the level of the amygdala.

Our findings have implications for treatment of anxiety disorders. Theories of emotion arguing for a central fear state^36^ and advocates of preparedness theory^37^ offer compelling evidence for conditioning as a translational model for development of stress and anxiety, and extinction as an analogue to exposure therapy for the treatment of anxiety disorders. However, these models are not in line with our discoveries on innate and conditioned threat. Our findings suggest that fear processes can work in parallel, and that treatment strategies should not only be based on studies of extinction of conditioned fear. This view mirrors the ideas of authors that have argued that current fear models are too focused on associative learning, and that this restrictive focus at most provides partial insight of fear processes in the brain^38^. These authors reason that stress and anxiety disorders are a result of complex interactions between innate and conditioned fear as well as risk-assessment and decision-making strategies. A way forward then, would be to examine fear processes not only in a relational sense, but also in which way this interaction shapes behavioral responses in risky situations that require adaptive action. This would be valuable for tackling avoidance behaviors, which are severely debilitating in all anxiety disorders.

Although our results suggest a parallel autonomic process for proximal and conditioned threat, alternative explanations must be considered. Given that most people are not subjected to immersive virtual-reality head-mounted displays in their daily life, it is possible that the results we observed are the cause of novelty and excitement, thus elevating SCR. This would be in line with previous studies demonstrating higher emotional ratings associated with immersive display media^39^. However, we only observed an effect of display media on proximal threat and not on conditioned threat. If viewing stimuli in immersive display media would explain our results, we would have expected an effect on all stimuli displayed. Therefore, novelty cannot explain the result.

Another concern is that the visual angle in different display media may have differed, in turn affecting our results. One possibility is that stimuli displayed in the head-mounted display were larger than the same stimuli displayed on a computer monitor, and that size influences SCR. However, in a previous study of SCR to proximal threat^4^, we used two classes of stimuli varying greatly in size and found that the larger stimuli with a greater visual angle did not facilitate SCR compared to the smaller stimuli, when presented at proximal distance. Therefore, it seems that depth information, not size, explains our results in the present study.

In addition, the elevated SCR we observed to proximal virtual characters could be the result of an increased orienting response. Orienting responses are not necessarily a result of threat. For example, studies have demonstrated that SCR increase to non-threatening flashes of light^40^ and opening of the eyes^41^. There is also substantial evidence suggesting that the amygdala, a structure found to moderate SCR to conditioned threat, is important for regulating attention to visual stimuli^42^. Thus, the increased SCR to proximal presentations observed here could be the result of visually driven attention and not threat. However, there is also evidence suggesting that modulation of SCR during fear conditioning to emotional relevant content (i.e. angry faces) is only partially regulated by attention as SCR is increased to threat also following back-ward masking^43^. We have also previously reported increased fear-potentiated startle to proximal stimuli^1^. Eye-blink startle is a defensive response that is increased to aversive and decreased to appetitive stimuli. Therefore, the increase in fear-potentiated startle to proximal stimuli can be interpreted as increased intensity in the defensive response. Hence, it seems likely that the observed facilitation of SCR to proximal stimuli in the present study also, at least partially, is related to increased intensity of the defensive response.

It is important to note that there could still be other factors affecting the difference in result between head-mounted display and computer monitor than the ones suggested here, especially since the experience of wearing a head-mounted display and sitting in front of a computer monitor involves more than just differences in display quality. For example, to test the modulation of proximal threat by visual depth, future studies could implement variations in the visual field by probing information from only one eye or disable stereoscopic vision where applicable.

In conclusion, results suggest that the autonomic component of proximal and conditioned threat responses may depend on parallel processes as proximal but not conditioned threat responses were enhanced by immersive virtual-reality as displayed in a head-mounted display. These findings have implications for understanding the neurobiological underpinnings of innate and conditioned fear in humans.

## Methods

### Subjects

Eighty healthy adult volunteers (female = 47; mean age = 25.3 yrs; range = 19–43 yrs) provided written informed consent in accordance with the Uppsala Ethical Review Board Guidelines. Subjects were randomly assigned to a group viewing stimuli immersed in virtual reality through a head-mounted display (n = 40, female = 26) or a group viewing stimuli on a regular computer display (n = 40, female = 21). For each group, participants were further randomly assigned to a group that viewed stimuli in an outdoor (n = 20) or indoor (n = 20) virtual context (see Fig. 1). In total, there were four groups of participants randomized to the experimental factors Display (immersive virtual-reality head-mounted display, computer display) and Context (Indoor, Outdoor). The methods used in this study was approved and performed according to the guidelines and regulations of the Uppsala Ethical Review Board.

### Materials

#### Contexts

In order to test whether threat responses generalized to different contexts we conducted the experiment using two different virtual environments. The environments were created in Unity (version 4.6.2, Unity Technologies, San Fransisco, CA) and modeled an indoor and one outdoor 3-dimensional setting. The indoor virtual environment consisted of a room with four red brick walls, grey concrete roof, and a wooden floor. The outdoor environment depicted a naturalistic scene with a large green open landscape, and trees scattered in the background (see Fig. 1). Contexts and stimuli were presented in a first-person perspective through an Oculus Rift immersive virtual reality headset (Oculus VR, Irvine, CA) and a regular computer display manufactured by Dell Inc (Round Rock, TX) (See Fig. 1). To minimize nausea from movement when using a virtual reality headset, the participants were instructed to remain still during the experiment.

#### Stimuli

Four male 3-dimensional virtual characters served as CSs for all groups and were always displayed in front of the participants. Two CSs were presented at a distance of 0.3 m (Proximal) whereas the other two CSs were presented at a distance of 2.7 m (Distant). These distances were the same as in one of our previous studies^4^. One of the Proximal and one of the Distant CSs predicted a mild electric shock which served as the unconditioned stimulus (US) whereas the other two CSs served as a distance matched control cues (CS−). Reinforcement rate was 50% during conditioning. Which of the characters served as the CS+ and CS− was counterbalanced across participants in each group. In each experimental phase, the characters appeared for 6 s. An inter-trial interval (ITI) followed each CS, with no CSs present for 8-12 s. Participants were told prior to the experiment that they could learn to predict the US but were not told which characters served as CS+.

The shock serving as US was delivered to the subjects’ wrist via pre-gelled disposable snap electrodes (EL503, BIOPAC Systems, Goleta, CA), and was calibrated using an ascending staircase procedure so that the shocks were rated as ‘uncomfortable’ but not ‘painful’^44^. US duration was 16 ms. Shock delivery was controlled using the STM100C module connected to the STM200 constant voltage stimulator (BIOPAC Systems, Goleta, CA).

#### Stimulus presentation software

Stimuli were presented in the immersive virtual-reality head-mounted display and on the computer display by using custom built software for Unity game engine (version 4.6.2, Unity Technologies, San Francisco, CA). The computer running the stimulus presentation software communicated with BIOPAC through Stimtracker (Cedris Corporation, San Pedro, CA) hardware.

#### Procedure

Participants were assigned to either use head-mounted display or computer display and then to one of the two context groups (Indoor and Outdoor) (see Fig. 1). For all experimental phases, stimulus order was pseudorandomized such that not more than two consecutive CS+ or CS− presentations occurred. Four stimulus presentation orders were used to counterbalance CS order across subjects. For each experimental group, conditioned stimuli (CSs) were presented in the same context before, during and after conditioning.

Before fear conditioning (habituation), each CS was presented four times. During this phase no fear learning had yet occurred. During fear conditioning (acquisition), each CS-type (CS+ and CS−) at each distance (Proximal and Distant) was displayed 8 times. Four of the CS+ presentations at the proximal location and four of the CS+ presentations at the distant location co-terminated with presentation of the US (50% partial reinforcement schedule). A test phase followed fear conditioning where each of the four CSs was presented 10 times without shock presentations (see Fig. 1). SCR during the conditioning phase and the following test phase were averaged over early (trials 1–5) and late trials (trials 6–10) to characterize the gross dynamics of SCRs within each phase^44^. There was a delay of 1 min between the conditioning phase and the following test phase.

#### Skin conductance responses

Skin conductance recording was controlled with the MP-150 BIOPAC system (BIOPAC Systems, Goleta, CA). Disposable Ag/AgCI SCR electrodes were placed on the palmar surface of the left hand. The signal was high-pass filtered at 0.05 Hz and SCRs were analyzed using Ledalab software package^45^ implemented in Matlab (Mathworks, Inc., Natick, MA). SCR were computed using the maximum phasic driver amplitude (Max.SCR) 1–4 seconds after CS presentation for each participant. SCRs were square root transformed to reduce extreme values and range-corrected by dividing all SCRs for each participants with each participants maximum SCR^2,46,47^. This way, SCRs ranged from 0 to 1.

### Statistical analysis

Repeated measures analysis of variance (ANOVA) and t-test implemented in SPSS (version 22, IBM Corporation, NY), was used to test the hypotheses. CS-type (CS+, CS−) and Proximal threat (Proximal, Distant) was modeled as within-subject factors and Contexts (Indoor, Outdoor) as well as Display type (immersive virtual-reality head-mounted display, computer display) as between subject factors. To evaluate extinction of conditioned threat and habituation of proximal threat, we modeled Time by splitting all trials in one early and one late phase. The number of trials for before, during and after conditioning were 2, 4 and 5 for each early/late phase respectively. The threshold for statistical significance was set to *p* < 0.05.

## Supplementary information

Supplemental material

## Data Availability

The datasets generated and analyzed during the current study are available from the corresponding author on reasonable request.
