# Supplementary material for: The effect of immersive virtual reality on proximal and conditioned threat
Source: Sci Rep. 2019 Nov 22;9:17407. doi: 10.1038/s41598-019-53971-z (PMC6874534; doi:10.1038/s41598-019-53971-z)
Supplement: Supplementary file 1 — Supplemental material [file 41598_2019_53971_MOESM1_ESM.pdf]

The effect of immersive virtual reality on proximal and conditioned threat

Jörgen Rosén[1*], Granit Kastrati[2], Aksel Reppling[1], Klas Bergkvist[1], Fredrik Åhs[3]

[1]Department of Psychology, Uppsala University, Uppsala, Sweden

[2]Department of Clinical Neuroscience, Karolinska Institutet, Stockholm, Sweden

[3]Department of Psychology, Mid Sweden University, Östersund, Sweden

Supplemental material

Results

Inhibition of proximal and conditioned threat

When including sex as a between subject variable in the ANOVA analysis we observed a higher statistically significant SCR activation for proximal compared to distant stimuli in the indoor context (Distance x Context: $F_{1, 80} = 5.68$, $p = .02$, $\eta^2 = 0.07$). We also observed higher activation for CS- distant presentations for the outdoor context when computer display was used (CS x Distance x Context x Display: $F_{1, 80} = 4.56$, $p = .04$, $\eta^2 = 0.06$).

Supplemental Table 1. Analysis of Variance (ANOVA) with conditioned stimulus type (CS+ and CS-), Context (Indoor and Outdoor), Distance (Proximal and Distant), Display (immersive virtual-reality head-mounted display and computer display) as factors during each phase.

	Before fear conditioning				During fear conditioning				After fear conditioning			
	df	F	η^2	*p*	df	F	η^2	*p*	df	F	η^2	*p*
CS	1,80	.10	.00	.75	1,80	21.92	.22	.00*	1,80	18.11	.19	.00*
CS x Context	1,80	1.06	.01	.31	1,80	.23	.00	.63	1,80	.00	.00	.98
CS x Display	1,80	2.13	.03	.15	1,80	.68	.01	.41	1,80	.98	.01	.33
CS x Context x Display	1,80	.03	.00	.87	1,80	1.76	.02	.19	1,80	1.29	.02	.26
Distance	1,80	178.92	.70	.00*	1,80	72.83	.49	.00*	1,80	40.74	.35	.00*
Distance x Context	1,80	.02	.00	.90	1,80	.48	.01	.49	1,80	3.17	.04	.08
Distance x Display	1,80	22.28	.23	.00*	1,80	11.35	.13	.00*	1,80	9.84	.12	.00*
Distance x Context x Display	1,80	2.27	.03	.14	1,80	1.11	.01	.30	1,80	1.68	.02	.20
CS x Distance	1,80	1.74	.02	.19	1,80	15.53	.17	.00*	1,80	.55	.01	.46
CS x Distance x Context	1,80	.03	.00	.88	1,80	.23	.00	.63	1,80	.33	.00	.57
CS x Distance x Display	1,80	.47	.01	.49	1,80	.54	.01	.46	1,80	.13	.00	.72
CS x Distance x Context x Display	1,80	1.98	.03	.16	1,80	.00	.00	1.00	1,80	4.56	.06	.04*

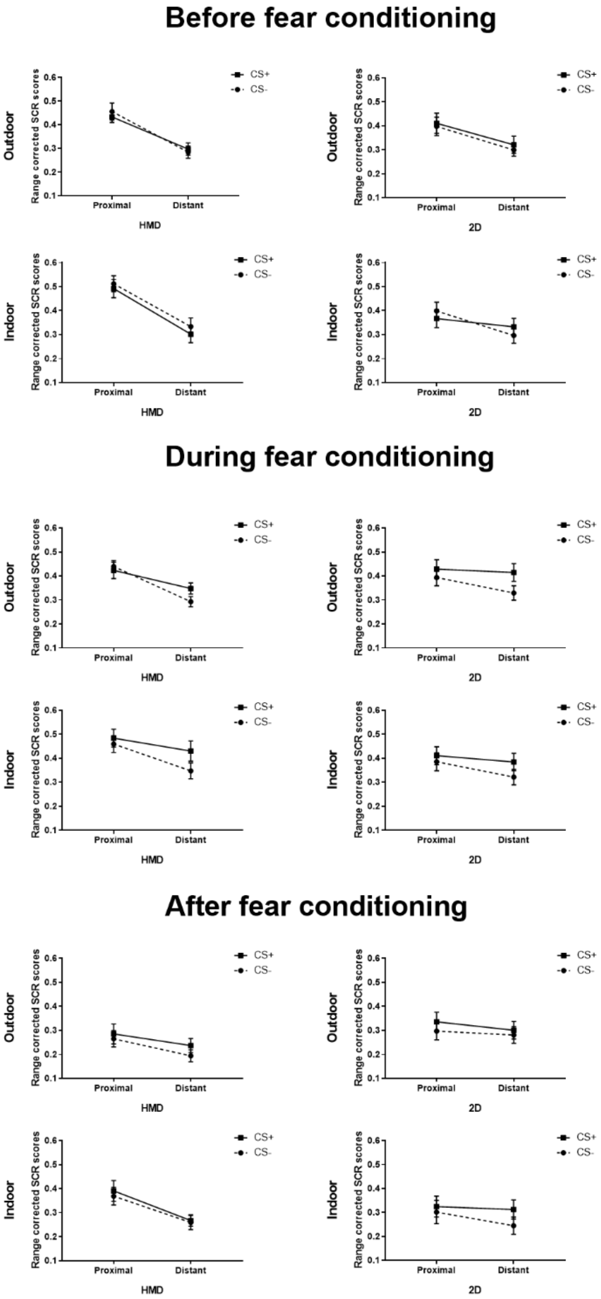

Supplemental Figure 1. Graphs depicts skin conductance responses (SCR) before, during and after fear conditioning. Stimuli presentations separated by Display (immersive virtual reality head-mounted display (HMD) and computer display (2D)) and Context (Indoor and Outdoor). Lines for each graph show CS+ and CS- activations for proximal and distant SCR.
